# Non‐model plants: Challenges and solutions

**DOI:** 10.1002/aps3.70074

**Published:** 2026-07-29

**Authors:** José M. Cunill‐Flores, Guanjing Hu, Aarón I. Vélez‐Ramírez

**Affiliations:** ^1^ Abess Center for Ecosystem Science and Policy, University of Miami Coral Gables Florida USA; ^2^ Shenzhen Branch, Guangdong Laboratory of Lingnan Modern Agriculture, Genome Analysis Laboratory of the Ministry of Agriculture and Rural Affairs, Agricultural Genomics Institute at Shenzhen Chinese Academy of Agricultural Sciences Shenzhen China; ^3^ Laboratorio de Ciencias Agrogenómicas, ENES León Universidad Nacional Autónoma de México, León Guanajuato México

Model organisms have fundamentally advanced plant biology. Research in a limited number of systems has revealed core principles of development, physiology, genetics, ecology, and evolution, and these systems continue to provide a critical foundation for discovery. The plant kingdom, however, is significantly more diverse than the subset of species with established genomic, experimental, and analytical resources. Many traits that require urgent explanation, such as unusual life histories, distinctive metabolic pathways, complex genome architectures, specialized ecological interactions, and responses to rapidly changing environments, are most evident in organisms that remain outside conventional model systems.

The term “non‐model” often suggests a lack of reference genomes, standardized protocols, transformation systems, curated databases, or widely adopted analytical pipelines. However, research on non‐model plants is not solely defined by these absences. These systems provide opportunities to test the generality of principles inferred from model organisms, to discover biological solutions that have evolved independently or uniquely, and to link methodological innovation to applied needs in conservation, restoration, agriculture, and environmental monitoring.

Research in non‐model systems reminds us that methods developed for model organisms cannot always be transferred directly. Protocols that work well in standard model species may fail when researchers are working with difficult tissues, degraded DNA from herbarium specimens, unusual morphologies, aquatic habitats, complex life cycles, polyploid genomes, or groups with very little comparative information. These challenges go beyond technical limitations; they also affect our mindset. If we become too dependent on the categories and assumptions that come from model systems, we risk overlooking important observations or drawing narrow conclusions.

This special issue, “Non‐model plants: Challenges and solutions,” addresses the methodological and analytical constraints encountered when studying non‐model plants of diverse taxa. The contributions systematically examine the limitations intrinsic to research outside recognized model systems and present solutions that expand the experimental and analytical tools available for the community. Specifically, the studies presented here show that these limitations can be reduced by three principal strategies: first, by adapting experimental and analytical methods when canonical assumptions derived from model organisms do not hold in novel phylogenetic or ecological contexts (Anaya et al., 2026; Garcia et al., 2026; Loew‐Mendelson et al., 2026); second, by developing and validating protocols that increase the accessibility and reduce costs of genomic and functional research (Anaya et al., 2026; Bullock et al., 2026; Gaischuk et al., 2026; Garcia et al., 2026); and third, by constructing analytical tools that explicitly integrate phenomic traits, ecological parameters, and management objectives (Weitkämper et al., 2026; Xie et al., 2026). Collectively, these advances include the development of protocols, comparative methodological assessments, open‐source analytical platforms, and cost‐reduction strategies, thereby enabling a broad range of research questions in genomic characterization, high‐throughput phenotyping, functional biology, applied ecology, and biodiversity assessment.

Loew‐Mendelson et al. (2026) examine the methodological challenges characteristic of studying photosynthetic diversity among freshwater submerged vascular plants. While aquatic plants exhibit photosynthetic diversity comparable to that of terrestrial species, several environmental factors in submerged habitats differ substantially. For example, slow diffusion of carbon dioxide in water, diel fluctuations in dissolved carbon dioxide, bicarbonate availability, and the absence or reduction of functional stomata complicate the direct application of methods designed for terrestrial leaves. Additionally, aquatic plants may possess physiological and metabolic adaptations that do not conform to terrestrial definitions of C_3_, C_4_, and Crassulacean acid metabolism (CAM) photosynthesis, increasing the chances that carbon‐concentrating mechanisms are misclassified, underestimated, or overlooked. To address these challenges, the authors review current physiological, biochemical, and molecular approaches for detecting carbon‐concentrating mechanisms in submerged vascular plants and highlight their strengths and limitations. The authors also go beyond a traditional review by presenting three practical experimental examples, including aquatic gas exchange measurements, phosphoenolpyruvate carboxylase (*PEPC*) gene expression assays, and diel assessments of acidity and organic acid accumulation. Through this approach, Loew‐Mendelson et al. provide a framework that integrates multiple lines of evidence and enables the characterization of aquatic photosynthesis based on the unique attributes of submerged plants, rather than imposing terrestrial categories.

Spatially explicit gene expression data are essential for elucidating the genetic mechanisms underlying plant development; however, traditional mRNA in situ hybridization often requires extensive optimization and presents particular challenges in complex or chemically recalcitrant tissues. Anaya et al. (2026) elegantly extend the use of a branched DNA probe‐based in situ hybridization method across distantly related non‐model species. The authors optimize the RNAscope assay (Advanced Cell Diagnostics [ACD], Hayward, California, USA) for reproductive tissues of *Mimulus lewisii* Pursh and subsequently apply the protocol to reproductive tissues of two gymnosperms, *Taxodium distichum* (L.) Rich. and *Juniperus virginiana* L., without further species‐specific adjustments. They also detect expression of *CYCLOIDEA* transcription factors in *M. lewisii* reproductive tissues, observing patterns consistent with previous studies. By demonstrating that a sensitive and streamlined assay can be applied across diverse reproductive tissues and taxa, this work expands the methodological toolkit available for developmental and functional genomics in both emerging model and non‐model plant systems.

Garcia et al. (2026) tackle another fundamental gap in plant functional genomics: the absence of a method for manipulating gene expression in gymnosperms, the sister clade to angiosperms. Using the xerophytic shrub *Ephedra tweedieana* Fisch. & C.A. Mey., which has a relatively compact genome and short generation time for a gymnosperm, they implement virus‐induced gene silencing (VIGS) via *Agrobacterium*‐mediated vacuum infiltration of tobacco rattle virus constructs targeting the *PHYTOENE DESATURASE* ortholog. The treatment produced a 64% silencing rate, with photobleaching visible within 10 days and persisting for at least five months across multiple tissue types. This represents the first successful application of VIGS in any non‐flowering plant, establishing *Ephedra* as a tractable system for investigating gene function in an evolutionarily critical seed plant lineage.

Long‐read (or third‐generation) sequencing technologies provide insights into genome structure, sequence variation, and epigenetic features, but the approach requires long, intact DNA molecules. Mature trees and wild‐collected non‐model plants frequently contain secondary metabolites and other endogenous contaminants that hinder DNA extraction and subsequent sequencing. In this issue, Gaischuk et al. (2026) optimize and ground‐truth a robust approach to increase the feasibility of high‐quality DNA in challenging taxa. The authors introduce an optimized, cost‐effective protocol that integrates nuclei purification, CTAB‐based lysis, organic solvent and alcohol clean‐up, and magnetic bead purification to obtain high‐molecular‐weight DNA suitable for Oxford Nanopore Technologies and PacBio sequencing. This method was validated across a range of taxa, including six *Nothofagus* Blume species, Andean–Patagonian gymnosperms, commercially important conifers, and model plants, consistently yielding high‐purity DNA fragments of at least 30 kbp. The protocol provides an accessible approach to long‐read sequencing for laboratories working with challenging plant tissues and limited resources.

One of the most persistent obstacles to genomic research in non‐model plants is cost, particularly for large‐scale studies. Population‐ and taxon‐scale genetic data are essential for conservation, phylogenetics, taxonomy, and studies of global change, yet the expense of generating large genomic datasets often restricts their widespread use, particularly in conservation projects and resource‐limited research groups. To reduce this barrier, Bullock et al. (2026) develop and validate a miniaturized library preparation protocol that leverages automated liquid‐handling robots to reduce reaction volumes to one‐tenth of standard preparations. Employing Angiosperms353 target capture across hundreds of DNA samples spanning a wide range of angiosperm families, from both fresh and silica‐dried herbarium tissues, they compare 0.5× and 0.1× reaction volumes across multiple quality metrics, including fragment size distributions, read mapping rates, and gene recovery. Although the number of libraries directly compared between the two volume conditions was limited, the consistent performance across the much larger set of independently prepared libraries supports the robustness of the miniaturized approach. The 0.1× volume libraries produce results that are generally comparable to those from 0.5× volume libraries, demonstrating that substantial cost reduction can be achieved without meaningful loss of data quality. Their findings support miniaturization as a practical strategy to expand access to population‐level sequencing, enhance the utility of herbarium collections, and democratize genomic approaches for non‐model taxa by lowering the financial threshold for participation in target‐capture studies.

Xie et al. (2026) address a major challenge at the intersection of non‐model systematics, restoration, and applied ecology: reliably distinguishing native and non‐native look‐alike oaks in the Pacific Northwest of North America. *Quercus garryana* Douglas ex Hook., the Oregon white oak, is the sole native oak throughout much of this region and serves as a keystone species in threatened oak ecosystems. In contrast, the European *Quercus robur* L. has been introduced as an ornamental, and it is often confused with *Q. garryana*, especially in young trees that lack distinctive bark, crown, and acorn characteristics. Misidentification may undermine restoration efforts by inadvertently introducing non‐native material into habitats intended to support native oak forests. To address this issue, Xie et al. integrate herbarium‐based leaf morphometrics, digital image analysis, Lasso logistic modeling, and software development to create Garryanalyzer, an open‐source ImageJ plug‐in that quantifies leaf traits and predicts species identity. The authors show that the tool demonstrated good performance with herbarium specimens and accurately identified *Q. robur* individuals sampled in Portland, while describing its assumptions and limitations. In addition to its immediate utility for land managers and restoration practitioners, Garryanalyzer offers a transferable workflow for developing accessible, taxon‐specific phenotyping tools in other non‐model systems.

Applied ecology frequently depends on observational datasets that are small, heterogeneous, labor‐intensive to collect, and shaped by complex environmental and historical factors. These characteristics complicate causal interpretation, particularly when conservation questions necessitate integrating knowledge from hydrology, geomorphology, land management, and related disciplines. To address this challenge, Weitkämper et al. (2026) present a conceptual and analytical framework for ecological datasets by applying Pearl's causal framework (Pearl, 2000) to assess factors influencing *Myricaria germanica* (L.) Desv. in northern Italy. The authors demonstrate how causal diagrams can clarify assumptions, structure variables, and inform the application of classical statistical models. In their case study, river channel width and riverbank protections are identified as key factors affecting juvenile survival, while other variables, such as altitude and human activity, exert influence through channel width as a mediator. This contribution underscores the value of transparent analytical frameworks in non‐model plant research, facilitating the transition from correlation to decision‐relevant causal hypotheses.

The studies in this issue emphasize the role of biological collections, field‐based observational expertise, and specific management requirements in performing research on non‐model organisms. Herbarium specimens, ex situ living collections, and in situ natural areas function not only as repositories of biological material but also as essential infrastructures for hypothesis formulation and testing across multiple temporal, spatial, and taxonomic scales. When these resources are systematically integrated with appropriate analytical tools, including data‐linkage tools and trait‐environment modeling methods, they enable the synthesis of historical and contemporary datasets, facilitate mechanistic interpretation of organismal traits within ecological contexts, and support the development of evidence‐based strategies for conservation and ecosystem restoration. Within this issue, such integration is achieved through adapting experimental protocols developed for systems with divergent biological assumptions, the redesign of workflows to minimize technical and financial limitations, and the implementation of open, reproducible, and directly applicable analytical tools. For non‐model taxa, this multidimensional integration is essential, as dependence on a single data modality or methodological approach is unlikely to yield comprehensive or generalizable insights.

As plant scientists confront accelerating biodiversity loss, climate crisis, habitat alteration, biological invasions, and growing demands for sustainable biotechnology, the capacity to work effectively in non‐model systems will become increasingly vital. Future methodologies must be robust to biological complexity, affordable for widespread adoption, and sufficiently open to allow modification for new organisms and research questions. This special issue aims to encourage researchers to view non‐model systems not as exceptions to established rules, but as catalysts for methodological innovation and biological discovery.

## AUTHOR CONTRIBUTIONS

J.M.C.F. wrote the first draft of the introduction, and G.H. and A.I.V.R. contributed text, edited the introduction, and approved the final version. J.M.C.F., G.H., and A.I.V.R. reviewed initial proposals and handled the manuscripts contributed to this special issue.
